# Non-inferiority Assessment of Maternal Adherence to Supplements, a Trial on the Effects of Multiple Micronutrient Supplementation (NAMASTE-MMS) in Nepal: study protocol

**DOI:** 10.1186/s13063-025-09031-1

**Published:** 2025-11-13

**Authors:** Kenda Cunningham, Sasmita Poudel, Mai-Anh Hoang, Aman Sen Gupta, Ramesh Adhikari, Dirghayu K.C., Diwakar Mohan, Yashodhara Rana, Bibek Kumar Lal, Lila Bikram Thapa, Rolf Klemm

**Affiliations:** 1 Helen Keller Intl, New York, USA; 2 Helen Keller Intl, Kathmandu, Nepal; 3 Johns Hopkins Bloomberg School of Public Health, Baltimore, USA; 4 Eleanor Crook Foundation, Washington D.C., USA; 5 Ministry of Health and Population, Kathmandu, Nepal

**Keywords:** Adherence, Acceptability, Antenatal, Postnatal, Iron and folic acid, Multiple micronutrient supplementation, Pregnancy, Post-partum, Trial, Nepal

## Abstract

**Background:**

Micronutrient deficiencies among pregnant women contribute to adverse maternal and neonatal outcomes. Multiple micronutrient supplementation (MMS) has proven its superiority when compared to the standard iron and folic acid (IFA) supplementation for maternal and infant morbidity and mortality. The Government of Nepal is exploring the scale-up of MMS, but first requires evidence such as on its adherence and acceptability. The objective of this study, thus, is to generate this needed evidence.

**Methods:**

The Non-inferiority Assessment of Maternal Adherence to Supplements, a Trial on the Effects of Multiple Micronutrient Supplementation (NAMASTE-MMS) in Nepal study is a three-arm, parallel, non-inferiority cluster-randomized controlled trial (c-RCT) assessing how well pregnant women adhere to and accept different types of supplements: MMS in blister packs or bottles versus IFA in blister packs. In one of Nepal’s seven provinces, Lumbini, the longitudinal NAMASTE-MMS study is being implemented across 120 health facilities (clusters), enrolling 2640 pregnant women into one of three arms: IFA-blister, MMS-blister, or MMS-bottle. The primary outcome is adherence to 180 supplements during pregnancy, measured by tablet counts with a non-inferiority margin of 13%. Secondary outcomes include comparisons of adherence between the two MMS arms and utilization of Antenatal Care (ANC), both potentially impacted by type of packaging. Exploratory outcomes include comparisons of adherence as well as the degree of acceptability to supplementation during early and mid pregnancy and post-partum.

**Discussion:**

Evidence generated from this study and three related mixed-methods implementation research studies will help the government in its potential scale-up of MMS supplementation during pregnancy and lactation.

**Trial registration:**

NCT06327646 (ClinicalTrials.gov, March 18, 2024 registered).

## Administrative information

Note: the numbers in curly brackets in this protocol refer to SPIRIT checklist item numbers. The order of the items has been modified to group similar items (see http://www.equator-network.org/reporting-guidelines/spirit-2013-statement-defining-standard-protocol-items-for-clinical-trials/).

**Table:** 

Title {1}	Non-inferiority Assessment of Maternal Adherence to Supplements, a Trial on the Effects of Multiple Micronutrient Supplementation (NAMASTE-MMS) in Nepal: Study Protocol
Trial registration {2a and 2b}.	ClinicalTrials.gov NCT06327646
Protocol version {3}	January 8, 2025, Version 4
Funding {4}	This study is funded as part of a grant from the Eleanor Crook Foundation. The MMS provided in bottles for the study were provided as in-kind support from Kirk Humanitarian.
Author details {5a}	Kenda Cunningham, Helen Keller Intl, USA Sasmita Poudel, Helen Keller Intl, Nepal Mai-Anh Hoang, Helen Keller Intl, USA Aman Sen Gupta, Helen Keller Intl, Nepal Ramesh Adhikari, Helen Keller Intl, Nepal Dirghayu K.C., Helen Keller Intl, Nepal Diwakar Mohan, Johns Hopkins Bloomberg School of Public Health, USA Yashodhara Rana, Eleanor Crook Foundation, USA Bibek Kumar Lal, Ministry of Health and Population, Nepal Lila Bikram Thapa, Ministry of Health and Population, Nepal Rolf Klemm, Helen Keller Intl, USA
Name and contact information for the trial sponsor {5b}	Yashodhara Rana Eleanor Crook Foundation Director- Research, Policy and Scaling (603) 667–8277 www.eleanorcrookfoundation.org yrana@eleanorcrookfoundation.org
Role of sponsor {5c}	The PI, Co-PI and research team have led the study design and data collection and will continue to make final decisions related to data management, analysis, interpretation of findings, writing up of results and submission of results for publication. The study sponsor and other stakeholders are collaborating but do not have ultimate authority over any of these activities.

## Introduction

Micronutrient deficiencies in pregnant women significantly increase the risk of maternal mortality and morbidity, and result in poor birth outcomes and higher rates of impaired cognitive and physical development in their children [1]. In Nepal, 12% of newborns are born with low birth weight (LBW) and 9% are born prematurely [2]. The maternal mortality rate stands at 151 per 100,000 live births [3], while the neonatal mortality rate is 21 per 1,000 live births [2]. Micronutrient deficiencies remain pervasive in Nepal, exacerbating these challenges. According to the 2016 Nepal Micronutrient Survey, 14% of pregnant women suffer from iron deficiency, with 34% of women of reproductive age (WRA) (15–49 years) being anemic [2]. Zinc deficiency affects 61% of pregnant women and 21% of children aged 6–59 months. Other prevalent deficiencies during pregnancy include Vitamin B6 (40%), Vitamin B12 (24%), Vitamin B2 (32%), Vitamin E (25%), Vitamin D (14%), and Vitamin A (7%) [4].

Taking a multiple micronutrient supplement (MMS) daily during pregnancy, based on the United Nations International Multiple Micronutrient Antenatal Preparation (UNIMMAP) formula, has demonstrated substantial benefits, including significant reductions in LBW, ), small-for-gestational-age babies, and 6-month mortality rates [5, 6]. In 2020, the World Health Organization (WHO) updated its guidelines, recommending MMS for pregnant women in the context of rigorous research [7]. Following this, in October 2021, the UNIMMAP-MMS formulation was added to the WHO Essential Medicines List (EML) as an antenatal supplement for pregnant women [8].

Evidence from Nepal supports the potential benefits of MMS. A randomized controlled trial conducted in 2002 in Dhanusha found that children born to mothers who took MMS during pregnancy, compared to those born to mothers who took only iron and folic acid (IFA), had higher birth weights, with these differences persisting until the children reached 2.5 years [9]. Furthermore, modeling studies have demonstrated the cost-effectiveness of MMS over IFA programs in Bangladesh, India, Pakistan, and Nepal [10, 11]. In 2023, Helen Keller Intl Nepal, with funding from the Vitamin Angel Alliance, collaborated with Nepal’s Ministry of Health and Population (MoHP) to conduct a landscape analysis to assess the enabling environment for UNIMMAP-MMS. This included a desk review of Nepal–specific scientific articles, reports, and publications; stakeholder consultations; and a workshop among key stakeholders—including government bodies, professional associations, United Nations agencies, and non-governmental organizations to build consensus on transitioning from IFA to MMS. Stakeholders identified the need for implementation research to assess the feasibility of the transition within Nepal’s healthcare system, as well as the acceptability, adherence, and cost-effectiveness of MMS.

In response, Helen Keller, in collaboration with the MoHP and the Eleanor Crook Foundation (ECF), agreed to carry out a set of implementation research studies that will inform the development of Nepal’s MMS scale-up strategy. One such study is a cluster randomized trial entitled Non-inferiority Assessment of Maternal Adherence to Supplements, a Trial on the Effects of Multiple Micronutrient Supplementation (NAMASTE-MMS) in Nepal.

### Objectives {7}

The primary aim of the NAMASTE-MMS study is to support the Government of Nepal to develop an evidence-informed strategy for the scale-up of MMS during ANC and PNC. The two primary research questions the trial addresses are as follows: (1) Is adherence among women during pregnancy to 180 MMS tablets non-inferior to adherence to 180 IFA tablets, both provided in blister-packs? and (2) Is adherence among women during pregnancy to 180 MMS tablets, packaged in a bottle, non-inferior to adherence to 180 IFA tablets, in blister packs? Other research questions include whether adherence to MMS tablets (both blister and bottle packaging) is non-inferior to adherence to IFA tablets (blister packs) at early, mid, and late pregnancy and post-partum; whether there is a difference in adherence to 180 tablets of MMS based on their packaging (blister versus bottle) during pregnancy; whether there is a difference in pregnant women’s uptake of antenatal care (ANC) based on MMS packaging and related quantity, frequency, and timing of pill distribution; and the degree of acceptability of IFA and MMS at different stages of pregnancy and in the post-partum period.

### Trial design {8}

This study is a three-arm cluster-randomized controlled trial (c-RCT) to compare adherence and acceptability of MMS and IFA supplementation. Pregnant women will be assigned to one of three trial arms (detailed in the intervention description). Data collection will occur through in-person surveys conducted at participants’ homes. Key data collection points include initial enrollment (following the pregnant woman’s recruitment at antenatal care between 12 and 13 completed weeks of pregnancy) and follow-ups at 30 and 90 days post-enrollment, after delivery, and at 45 days post-partum.

## Methods: participants, interventions, and outcomes

### Study setting {9}

Lumbini, the third largest province in Nepal by area and population with 5,122,078 inhabitants, was purposively selected as the c-RCT province due to its logistical feasibility and the presence of communities across all three of Nepal’s agro-ecological zones (AEZ): mountains, hills, and *terai* (plains). This diversity ensures valuable insights into how geographical, cultural, and ecological variations influence implementation as well as study outcomes. Health facilities (clusters) were randomly sampled to proportionally represent, as closely as possible, the population distribution across these zones—10% from mountains, 40% from hills, and 50% from *terai*. Using the list of government primary health care centers (PHCCs) and health posts (HPs), the required facilities (*n* = 120) were randomly selected and assigned to one of the three c-RCT arms. Women enrolled into the c-RCT from a particular facility were automatically assigned to that facility’s designated trial arm. Although health workers initially recruit pregnant women into the study, enrollment and data collection take place at the homes of the pregnant and lactating women, only after informed consent has been given.

### Eligibility criteria {10}

To be eligible for inclusion in the NAMASTE-MMS c-RCT, health facilities must meet the following criteria:

1. Be public/government-run;
2. Operate as a primary PHCC or HP at the time of randomization;
3. Implement the Government of Nepal’s standard ANC package of care; and.
4. Fall within the top 50% of average monthly caseloads for newly enrolled pregnant women (i.e., first ANC) for their respective agro-ecological zone (mountains—at least 5 new cases/month; hills—at least 8 new cases/month; and terai—at least 15 new cases/month) based on health management information system (HMIS) ANC data (July 17, 2022, to July 16, 2023).

To be eligible for inclusion in the NAMASTE-MMS c-RCT, pregnant women must meet the following criteria:

1. Be between 18 and 35 completed years of age at the time of health facility enrollment;
2. Not have plans to move or migrate for at least 9 months after enrollment;
3. Have a gestational age of at least 12 but not yet 14 weeks at time of health facility enrollment, estimated based on her last menstrual period (LMP) and confirmed by enumerators during their initial visit;
4. Be experiencing a healthy pregnancy without complications and not classified as severely anemic or high risk. Exclusion criteria include conditions such as diabetes, polycystic ovary syndrome, thyroid disease, kidney diseases, fibroids, and severe mental/physical conditions that prevent study participation (e.g., inability to speak, attend ANC/PNC, take supplements, or cognitive impairments);
5. Provide written informed consent to participate in the study; and
6. Agree to have enumerators visit the home at different data collection points.

### Who will take informed consent? {26a}

Enumerators initially obtain informed contact when they visit the pregnant woman at her home for enrollment in the study, following ethical norms. Enumerators again collect each woman’s written informed consent at each of the four subsequent data collection points. Participants are free to withdraw at any time, for any reason without consequence and will be made aware of this during each informed consent process.

### Additional consent provisions for collection and use of participant data and biological specimens {26b}

Not applicable—No provisions are required for collection or use of participant data and no biological specimens will be collected in the NAMASTE-MMS c-RCT.

## Interventions

### Explanation for the choice of comparators {6b}

In Nepal, government health services routinely provide IFA supplements to women during pregnancy and the post-partum period. The standard regimen is one daily tablet containing 60 mg of elemental iron and 400 µg of folate, beginning at the start of the second trimester of pregnancy and continuing for 45 days postpartum, totaling 225 days of supplementation [12]. Despite its routine use, adherence to IFA has been suboptimal, with a self-reported adherence rate of approximately 65%.

Each MMS tablet, based on the UNIMMAP formulation, contains 15 micronutrients: 30 mg of elemental iron, 400 µg of folic acid, 800 µg of vitamin A, 200 IU of vitamin D, 10 µg of vitamin E, 70 mg of vitamin C, 1.4 mg of thiamin, 1.4 mg of riboflavin, 18 mg of niacin, 1.9 mg of vitamin B6, 2.6 µg of vitamin B12, 2 mg of copper, 150 µg of iodine, 65 µg of selenium, and 15 mg of zinc [13].

The value of MMS over IFA for maternal and child health has been documented in several studies. Implementation research, however, is essential to assess context-specific adherence and acceptability to MMS and barriers to its adoption, including any variation by packaging type (i.e., blister packs versus bottles).

### Intervention description {11a}

Each of the three arms of the NAMASTE-MMS c-RCT has a distinct supplementation approach:

1. *IFA-blister arm*: participants receive IFA tablets in blister packs. Distribution follows current national guidelines with women receiving: 60 tablets during the first ANC visit; 30 tablets during the second, third, fourth, and fifth ANC visits; and 45 tablets during the first PNC visit.
2. *MMS-blister arm*: participants receive MMS tablets in blister packs. Distribution mirrors the IFA arm, with women receiving: 60 tablets during the first ANC visit; 30 tablets during the second, third, fourth, and fifth ANC visits; and 45 tablets during the first PNC visit.
3. *MMS-bottle arm*: participants receive MMS tablets in bottles during pregnancy and blister packs during the post-partum period. Distribution involves women receiving: 90 tablets in a bottle during the first ANC visit; 90 tablets in a bottle during the third ANC visit; and 45 tablets in blister packs during the first PNC visit.

The blister pack for the 45 tablets given for postnatal supplementation in all three arms will be marked with a red or other brightly colored marker so that both study participants and enumerators can easily differentiate their pregnancy and post-partum supplements.

Eligible pregnant women are identified during ANC visits by health workers, who document details such as the woman’s name, contact information, and address in an enrollment log. This log also tracks eligibility criteria to ensure proper screening. Women who meet the criteria are informed about the study objectives, procedures, and potential benefits, and initial verbal consent is obtained.

Enumerators then visit the women at home to confirm eligibility, obtain written informed consent, and administer an enrollment survey. The recruitment process is conducted on a rolling basis until the target sample size of 2640 women (approximately 22 per facility) is reached. Recruitment is expected to take 3 months in the *terai* and progressively longer in the hills and mountains due to lower population density and service usage.

Information and Education Communication (IEC) materials, tailored to each study arm (i.e., IFA vs. MMS), were designed to enhance understanding of supplements and support proper adherence. These materials include the following: (1) a job aid to support health worker counseling of pregnant women on the supplement they receive; (2) a poster for health facilities to display; and (3) a take home card for participants and their family members to discuss the supplementation. These materials provide guidance on supplement use, benefits, potential side effects, and contact information for further assistance.

### Criteria for discontinuing or modifying allocated interventions {11b}

If any unforeseen challenges occur at a participating health facility, such as serious violations of study protocol or ethics, or a facility no longer meets the NAMASTE-MMS c-RCT criteria, the research team would consult with relevant government officials, the Nepal Health Research Council (NHRC), and the external Trial Steering Committee (TSC) to inform its decision on whether or not to discontinue the facility in the study and to determine appropriate next steps.

For individual participants, if severe side effects potentially related to the trial supplements are reported (e.g., excessive vomiting, lethargy, or significant weakness), the participant will be referred to the health facility where they were enrolled into the trial to seek out clinical evaluation. Based on the health provider’s guidance, the participant may continue in the study or be withdrawn.

In the event of adverse pregnancy outcomes such as miscarriages and stillbirths, the participant will be excluded from further data collection.

Participants will also be discontinued if they are found to no longer meet the inclusion criteria during the study period. Examples include relocattion out of the study area or developing severe mental or physical conditions that prevent their participation. Reason for exclusions and related details will be recorded systematically to ensure transparency and accountability.

### Strategies to improve adherence to interventions {11c}

Health workers will continue to provide standard ANC and PNC services across all trial arms. The only variation will be the type of supplementation and related IEC materials. No additional adherence-enhancing strategies will be implemented to ensure that adherence to IFA and MMS—the trial’s primary outcome—is assessed without external influence. This approach ensures that the study reflects real-world conditions as closely as possible.

### Relevant concomitant care permitted or prohibited during the trial {11d}

Nepal’s national guidelines, including the National Strategy for the Control of Anemia Among Women and Children in Nepal [12], the ANC-to-PNC-continuum-of-care-guideline [14], and National Medical Standards for Maternal and Newborn Care – Volume III, 2020 [15], recommend daily IFA prophylaxis for all pregnant women and therapeutic supplementation for severe anemia (Hb < 7.9 g/dL). Health workers will follow current clinical guidelines as outlined in the ANC-to-PNC-continuum-of-care-guideline and National Medical Standards for Maternal and Newborn Care – Volume III, 2020 to identify and manage cases of anemia. Any participant with mild anemia will continue supplementation and any participant with moderate anemia will continue supplementation plus have a top-up of iron; any participant who develops severe anemia will be excluded from the trial, regardless of treatment protocol, trial arm, or other factors.

### Provisions for post-trial care {30}

All participants will continue receiving standard ANC and PNC services. No additional post-trial care will be provided.

### Outcomes {12}

#### Primary outcome

The primary outcome is the non-inferiority of adherence to 180 MMS tablets compared to 180 IFA tablets, based on pill counts. This primary outcome will compare the IFA-blister versus MMS-blister arms and the IFA-blister versus MMS-bottle arms and will be measured in two ways: (1) binary—whether the study participant consumed at least 180 supplement tablets between study enrollment and delivery and (2) continuous (0–180)—the number of supplements consumed between study enrollment and delivery.

#### Secondary outcomes

A secondary outcome is adherence to 180 MMS tablets, based on pill counts. This secondary outcome will be measured in the exact way as the primary outcome; the comparison this time will be between the MMS-blister and the MMS-bottle arms. Another secondary outcome to assess adherence to 180 MMS tablets compared to 180 IFA tablets, based on pill counts, incorporates each participant’s eligibility period to receive up to 180 tablets (e.g., early delivery). This secondary outcome will compare each of the arms to each other (IFA-blister and MMS-blister arms; IFA-blister and MMS-bottle arms; and MMS-blister and MMS-bottle arms) and will be measured in two ways: (1) binary—whether the study participant consumed at least the maximum number of supplement tablets eligible to consume between study enrollment and delivery and (2) continuous (0-number of eligible days)—the number of supplements consumed between study enrollment and delivery.

The secondary outcome of ANC utilization will involve comparison between the MMS-blister and MMS-bottle arms and will be measured in two ways: (1) binary—whether the study participant received the recommended 8 ANC visits during pregnancy and (2) continuous—the number of ANC visits the study participant received during pregnancy. This will be assessed by recording the information about ANC visits from the individual’s MNH card.

#### Exploratory outcomes

Adherence to supplementation for 30 days and 90 days post-enrollment during pregnancy and 45 days post-partum will also be assessed as an exploratory outcome. This outcome will compare each of the arms to each other (IFA-blister and MMS-blister arms; IFA-blister and MMS-bottle arms; and MMS-blister and MMS-bottle arms) and will be measured in two ways: (1) binary—whether the study participant consumed at least 30, 90, and 45 supplement tablets between study enrollment and the 30-day follow-up visit, 90-day follow-up visit, and 45-day post-partum follow-up visit, respectively and (2) continuous (0–30, 0–90, and 0–45)—the number of supplements consumed between study enrollment and the 30-day follow-up visit, 90-day follow-up visit, and 45-day post-partum follow-up visit, respectively.

IFA and MMS acceptability at 30 days, and90 days post-enrollment during pregnancy, delivery (~180 days), and 45 days post-partum will also be assessed. This exploratory outcome will compare each of the arms to each other (IFA-blister and MMS-blister arms; IFA-blister and MMS-bottle arms; and MMS-blister and MMS-bottle arms) and will be measured as a continuous variable (0–25), based on summing 5-point Likert scale data for each of five organoleptic aspects: taste, color, swallowability, odor, and dosing frequency.

### Participant timeline {13}

**Fig. 1:**
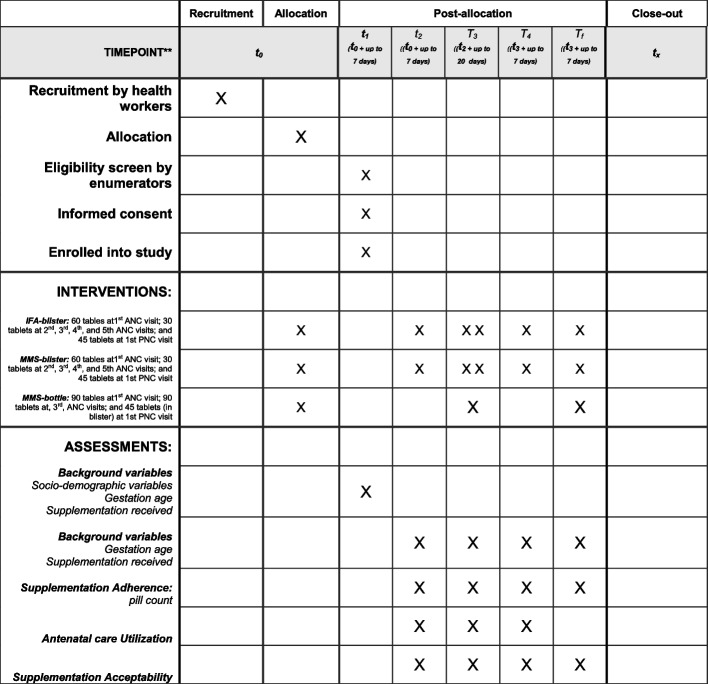
NAMASTE-MMS schedule of enrolment, interventions, and assessments throughout the study period

The enrollment, interventions, and assessments are identical across all trial arms. Recruitment by health workers during ANC visits involves providing the initial dose of supplementation to those pregnant women who meet the inclusion criteria and express interest in study participation. Then, enumerators conduct a home visit for formal study enrollment and informed consent. Once enrolled, participants are followed and interviewed at specific timepoints (30 and 90 days post enrollment, after delivery, and 45 days post-partum), contingent upon further consent. Pill distribution slightly varies by arm, as described above. Further details on the timeline and distribution schedule are illustrated in Fig. 1.

### Sample size {14}

The NAMASTE-MMS c-RCT is designed to test the non-inferiority of MMS adherence compared to current adherence to IFA (65%), with an anticipated adherence rate of 66% in each group. The sample size calculation was based on the following parameters:

- A non-inferiority margin of 13 percentage points
- An alpha (significance level) of 0.05
- An intra cluster correlation coefficient (ICC) of 0.1 at the facility level.

The sample size was calculated using the following formula:

$$\mathrm n\;=\;1\;+\;\mathrm f\left(\mathrm\alpha,\;\mathrm\beta\right))\;\ast\;(((\mathrm P1\;\ast\;(1-\mathrm P1))\;+\;(\mathrm P2\;\ast\;(1-\mathrm P2)))\;\ast\;(1\;+\;((\mathrm m-1)\ast\mathrm{rho})))\;/\;(\mathrm m\ast({(\mathrm P1-\mathrm P2-\mathrm d)}^2)))$$

where,

- *n*—number of clusters required
- P1—adherence in IFA arm
- P2—adherence in MMA arm
- *m*—number of pregnant women enrolled at each facility
- rho—intraclass correlation coefficient at facility level
- *d*—inferiority margin
- *α*—type I error
- *β*—type II error

To achieve 80% power in detecting non-inferiority using the lower-bound of the two-sided 97% confidence interval (CI) for the percentage difference in adherence proportions, and to account for a 15% loss to follow-up (due to withdrawal, miscarriage, stillbirths, or other factors), the required sample size was determined as follows:

- 880 pregnant women per arm (IFA-blister, MMS-blister, and MMS-bottle)
- 2640 pregnant women in total from 120 facilities (40 facilities per arm).

Statistical inference for non-inferiority will be performed using the lower bound of the 97% CI, with adjustments for multiple hypotheses using the Bonferroni correction. This approach ensures robust statistical conclusions while minimizing the risk of type I errors.

#### Recruitment {15}

Pregnant women, at least 12 and not yet 14 completed weeks of gestational age, who are attending their first antenatal care visit at one of the 120 health facilities in the study and who have not yet started consuming prenatal IFA or MMS are considered potential study participants. Health workers will begin by filling out an enrolment log, which includes the woman’s name and eligibility screening information:

- At least 12 but not yet 14 completed weeks of pregnancy.
- Between 18 years and 35 completed years of age.

If the pregnant women meet these two initial criteria, the health worker will document additional details, including phone number(s) for the woman and some of her other household members and house location details. The health worker will then inform the woman about the study, objectives, and procedures, as well as detailed information about the supplements including their timing, benefits, and potential side effects to ensure clarity prior to recruitment.

The enrollment log will serve as a comprehensive record of all pregnant women screened for the study, including those excluded, the reasons for exclusion, and any refusal to participate. For women who meet study inclusion criteria and agree to participate, the health worker will share their information with the enumerator assigned to that health facility. Enumerators will then proceed with the formal enrollment process, including confirming eligibility and obtaining written informed consent during a home visit.

## Assignment of interventions: allocation

### Sequence generation {16a}

The 120 facilities from 10 districts which met the selection criteria for adequate ANC visit volumes, as described above were selected. Among the 131 health facilities meeting study inclusion criteria, 103 were from *terai*, 21 from hills and 7 from the mountainous agro-ecological zones. Within the available facilities for each agro-ecological zone, 120 facilities were randomly selected with stratification by the agro-ecological zone using the randomizer package in R. The facilities were sampled to randomly allocate each to one of three groups: districts were used as blocks to ensure balance across zones. The final distribution among facilities is as follows: 93 from the *terai*, 21 from the hills, and 6 from mountains.

At a provincial kick-off meeting held in Lumbini with key stakeholders, government officials used a lottery system to allocate the three groups of heath facilities groups to the three study arms (IFA-blister, MMS-blister, or MMS-bottle). This participatory approach ensured transparency and stakeholder engagement in the allocation process.

### Concealment mechanism {16b}

Due to the nature of the intervention—delivery of supplements to pregnant and lactating women—concealment of allocation was not feasible.

### Implementation {16c}

At each study facility, all women were deemed eligible for inclusion or not during their first antenatal visit. After recruitment into the study by a health worker, the health worker notifies the enumerator who records the woman’s details in his/her electronic enrolment log. The enumerator then visits the woman at her home to verify her eligibility based on the inclusion and exclusion criteria and obtain written informed consent for study participation. For those women who meet the criteria and provide consent, the enumerator administers the enrollment survey during the same home visit.

The enumerators return to conduct follow-up survey visits with study participants at 30 days and 90 days post enrollment as well as at delivery and 45 days post-partum. If a pregnant woman is unavailable during the 30-day, 90-day, or 45-day post-partum data collection point for more than 7 days, she will be excluded from the interview process for that round. She will, however, remain a study participant and be approached again at subsequent data collection points. For the delivery data collection point, the timeframe for data collection will be extended, allowing data to be collected within 20 days of childbirth, but if unavailable for more than 20 days, she will be excluded from that round of data collection but approached again for the final 45-day post-partum data collection point.

## Assignment of interventions: blinding

### Who will be blinded {17a}

Study participants, researchers, and enumerators, and health workers recruiting pregnant women will not be blinded to treatment arm. All parties will be informed about whether participants are receiving IFA or MMS.

### Procedure for unblinding if needed {17b}

Not applicable—No blinding in the NAMASTE-MMS c-RCT.

## Data collection and management

### Plans for assessment and collection of outcomes {18a}

Enumerators will use semi-structured questionnaires to conduct in-person interviews as part of this longitudinal study of pregnant and lactating women. Data will be collected using mobile devices equipped with Open Data Kit (ODK) platform. After verifying all survey entries are complete, enumerators will submit finalized surveys at the end of each day. To maintain the rigor of assessments, enumerators receive comprehensive training and weekly follow-up supportive supervision. Data collection will occur at five key time points:

1. Study enrollment
2. 30 days post enrollment
3. 90 days post enrollment
4. Delivery (about 180 days post enrollment), and
5. 45 days post-partum

The data collection tools gather a range of demographic and pregnancy-related information, including socio-economic status, maternal age, age at marriage, parity, and gestational age, as well as supplement (IFA or MMS) adherence and acceptability; antenatal care visits and services received during ANC (or delivery and PNC as appropriate); knowledge of ideal practices during pregnancy and postpartum to promote maternal and child health and nutrition; intended place of delivery, and plans for relocation (i.e., to parental home or “Maiti”) before or after delivery, including duration; and other related factors.

These data collection tools were initially developed in English, then translated into Nepali and Awadhi (for some *terai* communities where Nepali is not the first language). A separate team performed back-translation into English to enhance data quality. All tools underwent pre-testing in the field, after being designed by a group of technical experts, and were subsequently refined before finalization and integration into the digital data collection system: ODK.

The electronic tools include features designed to ensure high-quality data collection: automatic range and consistency checks, verification of skip patterns, and built-in safeguards to disallow clearly erroneous responses. A flagging system has been implemented to address potential issues during data collection. For example, if a response indicates a serious violation of study inclusion criteria or intervention implementation (i.e., receipt of incorrect supplements, lack of counseling, or absence of a take home card), the system will alert the enumerator. Enumerators are provided with prompts on how to handle flagged cases including encourage the individual to seek healthcare, call the research officer to immediately visit the health facility, and contact the Kathmandu-based team to determine whether to drop the case from the trial.

### Plans to promote participant retention and complete follow-up {18b}

To ensure participant retention and complete follow-up, the study team will employ the following strategies: maintaining updated contact information, tracking participants, and appreciating participants.

To maintain updated contact information, the enumerators will collect and regularly update the contact details of enrolled pregnant women, including their address, and phone number(s), as well as regularly update the phone number(s) of the participant’s spouse or other household members. This information will enable communication throughout the study duration.

Enumerators will make every reasonable effort to track participants and maintain their engagement throughout the study. In cases of relocation, the impact on study participation will be assessed based on the following criteria:

1. Is the relocation within the same health facility catchment area? If a participant moves within the same catchment area, she will remain eligible to continue in the study.
2. Is the relocation outside the study catchment area? If a participant relocates to a new health facility catchment area not included in the study, she will be discontinued from participation.
3. Is the relocation for delivery or postpartum visits? If a participant moves temporarily (e.g., for delivery or Maiti visits), enumerators will coordinate to ensure she receives postpartum supplements, enabling continued participation.

Additionally, as a token of appreciation, each participant will receive Rs 100 mobile credit at every data collection point, irrespective of their study arm allocation. This incentive recognizes their time and effort in contributing to the study.

### Data management {19}

Data will be uploaded daily to a secure web-based platform. Study team members will have access to uploaded data to regularly review it to identify and correct errors. Data will be downloaded to check quality and consistency and provide timely feedback as needed. All corrections made to the data will be recorded. Data cleaning and verification will include translating any data (e.g., other (specify) responses) into English and reclassifying the responses into pre-existing and/or new response categories, where necessary, followed by standard data cleaning procedures such as checking ranges and skip patterns before starting the process of variable generation and tabulations. All data management, including variable generation, will be done using Stata, maintaining user-friendly log and do files for easy replication of data management steps.

### Confidentiality {27}

Security and confidentiality of participant data will be handled carefully throughout the research. All participant data will be anonymized, and each participant will be assigned a unique ID number to safeguard their identities effectively. To reinforce data security, password protection will be used at various levels. Data collection will be conducted through the password-protected ONA digital platform, while data will later be managed and analyzed in Stata (which are password-protected secure sites under management by Helen Keller standard of practice). All laptops which have access to this site are password protected and the data in the site is also password protected. All unattended devices will be kept in a securely locked room with restricted access. Only authorized individuals will have access to keys of these devices, minimizing the risk of unauthorized use.

### Plans for collection, laboratory evaluation and storage of biological specimens for genetic or molecular analysis in this trial/future use {33}

Not applicable as biological specimens will not be collected in this trial.

## Statistical methods

### Statistical methods for primary and secondary outcomes {20a}

For all primary and secondary outcomes, crude estimates along with their 95% confidence intervals, adjusted for clustering at the health facility level, will be presented for all 3 study arms.

Before analyzing treatment effects for primary and secondary outcomes, we will assess baseline comparability by allocation arm for socio-economic and demographic factors, as well as maternal health and pregnancy history variables, using data collected in the enrollment survey (prior to IFA or MMS provision). Differences in baseline characteristics will be examined by comparing means and proportions, but no statistical tests will be conducted for these comparisons, following Consolidated Standards of Reporting Trials (CONSORT) guidelines for effectiveness trials.

Categorical variables will be summarized using frequencies and percentages calculated based on the number of participants with non-missing data. Continuous variables will be displayed as mean and standard deviation (SD) or median and interquartile range, as appropriate, based on their distribution. These analyses aim to confirm balance between arms post-randomization. Any baseline characteristic with a difference of 10% or more will be considered for inclusion in adjusted models for the primary and secondary outcomes.

For non-inferiority analyses (e.g., the primary research question), treatment effect models will use a per-protocol approach, including all enrolled women with complete data on the primary outcome (180 adherence) who comply as per their study arm allocation (women who adhere to the “intervention” they are allocated).

Absolute differences in the proportion of women adhering to MMS (compared to IFA) will be calculated for each MMS arm, along with 95% confidence intervals. Results will be plotted and visualized using graphical methods. Non-inferiority will be established if the lower limit of the confidence interval is above the non-inferiority margin (i.e., 53%). Superiority will be determined if the lower limit of the confidence interval exceeds the hypothesized adherence estimate of IFA (i.e., 66%). The lower and upper limits of the confidence interval will be updated to the actual values of the control (IFA) arm.

Primary and secondary outcomes that are measured as binary variables (e.g., supplement adherence and ANC attendance) will be analyzed using log-binomial regression models with generalized estimating equations (GEE) to account for clustering at the health facility level. Log-binomial regression is selected in our context as the outcome is not expected to be rare; it directly estimates the relative risk and is a more interpretable and actionable result for clinicians and policy makers to use. Results will be presented as relative risk ratios and absolute risk differences. Primary and secondary outcomes measured as continuous variables (e.g., adherence to supplementation, adherence to ANC, and acceptability) will be analyzed using linear mixed models with GEE. Results will be reported as effect sizes (mean/DS or median/IQR, depending on distribution). Clustering at the health facility level will be accounted for in all GEE models.

To control the family wise error rate, a conservative Bonferroni correction will be applied. For the three-arm comparisons, the type-1 error threshold will be adjusted to a *α* = 0.0165. Estimates with 95% confidence intervals will also be provided to facilitate comparison across similar studies and outcomes.

### Interim analyses {21b}

The only interim analyses planned are to assess primary and secondary outcomes once at least 80% of data has been collected at each data point. These de-identified results would be shared only with Trial Steering Committee members, Government of Nepal stakeholders and the donor.

### Methods for additional analyses (e.g., subgroup analyses) {20b}

Not applicable as additional subgroup analyses are not planned. Any done as secondary analyses would be exploratory in nature.

### Methods in analysis to handle protocol non-adherence and any statistical methods to handle missing data {20c}

Enumerators will make every effort possible to collect data until the end of the study with each participant, unless she withdraws consent for data collection. Missing data for all variables including treatment and outcomes will be assessed. If less than 10% of observations are missing for the outcome variable in any of our models, we will use a complete case analysis and report the number of observations used in the analysis. If more than 10% are missing for the outcome variable in any of our models and this missingness is at random (as determined by the distribution of baseline variables), we will consider imputing outcomes using multiple imputation chained equations (MICE) [16]. Sensitivity analysis will be done with and without the imputed data. As part of sensitivity analysis, we will take the most conservative situation of considering enrolled women who are lost to follow-up as having an unfavorable adherence outcome, given that high drop-out rates are known to influence results for non-inferiority trials.

### Plans to give access to the full protocol, participant-level data, and statistical code {31c}

Upon reasonable request, the corresponding author will consider providing access to the full protocol, participant-level data, and statistical code.

## Oversight and monitoring

### Composition of the coordinating center and trial steering committee {5d}

Several oversight and monitoring processes are planned to ensure systematic monitoring for quality implementation. The Kathmandu-based research team supervise the work of the research officers in the ten study districts of Lumbini and research officers supervise the work of research associates/enumerators in their clusters as well as health workers and FCHVs; for this, supportive supervision protocols and related tools are used. Supervision visits intend to provide on-site coaching, verify that the trial protocol including ethics are being followed, and identify and correct any errors or inconsistencies in data collection.

Furthermore, as the intervention is reliant upon effective delivery of ANC and PNC, including supplementation distribution and counseling, a quality assurance system is also being put in place to monitor activities at the health facilities involved in the studies. Each health facility will be visited by a research officer as early as possible during the enrollment period and then as often as possible (at least once every 3 months) throughout the entirety of the data collection process. Similarly, FCHVs are a critical part of delivering health and nutrition services at the community level including sharing information on a 1:1 basis, facilitating group-based meetings, and informally being consulted for health-related advice. Thus, both research officers and enumerators will visit as many FCHVs as possible throughout the life of the project to ensure that health workers have shared appropriate information with FCHVs and that FCHVs in turn, are aware and comfortable with sharing information on supplementation.

The research sub-committee within the NuTEC committee under Family Welfare Division (FWD), MOHP will serve as the NAMASTE MMS Trial Coordination Committee (TCC). The members within the committee are responsible for providing technical review and inputs to the study design; to review semi-annual progress reports; to support coordination with relevant subnational stakeholders for smooth implementation of the trial; observe research implementation and provide feedback and recommendations to the study team; review and support interpretation of study findings; help facilitate the sharing of research findings with relevant stakeholders.

The NAMASTE MMS TSC will include a small team of experts responsible for providing technical review and inputs to the study design including review of study protocol; reviewing semi-annual progress reports; supporting trial implementation and providing any recommendations to the study team; reviewing and supporting interpretation of study findings; and sharing of research findings with relevant stakeholders. As of August 5, 2024, these experts include Dr. Bibek Kumar Lal, Director of FWD, MoHP; Mr. Lila Bikram Thapa, Section Chief, Nutrition, FWD, DoHS, MoHP; Dr. Parul Christian, Johns Hopkins University; Dr. Crystal Karakochuk, University of British Columbia; Dr. Martin Mwangi, Micronutrient Forum; Stephanie Suhowatsky, JHPIEGO; Dr. Naomi Saville, University College London; Dr. Kristen Hurley, Vitamin Angels; and Nadia Diamond-Smith, University of California, San Franciso.

### Composition of the data monitoring committee, its role and reporting structure {21a}

Not applicable as a data monitoring committee was not established for this trial.

### Adverse event reporting and harms {22}

In cases of adverse pregnancy outcomes such as miscarriages and still births, those women will be excluded from the study for further data collection and the reason of exclusion and details surrounding the event recorded. Also, participants will be discontinued if it is discovered that they no longer meet the study inclusion criteria (ex: moves out of the study area, develop a severe mental or physical condition that affects their study participation) during the trial period.

### Frequency and plans for auditing trial conduct {23}

There are no plans thus far for auditing trial conduct.

### Plans for communicating important protocol amendments to relevant parties (e.g., trial participants, ethical committees) {25}

The Principal Investigator and Co-Principal Investigator will take the lead responsibility for sharing relevant information and addressing any issues that may arise during the trial including notification of necessary protocol amendments, as appropriate, to the NHRC as well as the TSC, TCC, donor, government stakeholders, and participants.

### Dissemination plans {31a}

Emerging study findings will be communicated with the MMS research sub-committee and the FWD quarterly with the aim of providing valuable insights and evidence to inform the government’s plans for a strategy to scale-up MMS in Nepal. Furthermore, once all trial analyses are completed, dissemination events at the federal level and in Lumbini province will be organized involving all relevant stakeholders. Additionally, the findings will be disseminated globally with various stakeholders through presentations at academic conferences and other platforms, as well as publication of multiple research papers in peer-reviewed journals.

## Discussion

The NAMASTE-MMS study aims to address critical gaps in evidence regarding adherence and acceptability of multiple micronutrient supplementation (MMS) compared to iron and folic acid (IFA) in Nepal. The study design, which includes two distinct types of MMS packaging—bottle and blister packs—will provide valuable insights into how packaging influences maternal adherence and other outcomes. Such evidence is particularly relevant in settings like Nepal, where micronutrient deficiencies remain a significant public health challenge.

One challenge is the reliance on secondary data for study design parameters, including sample size calculations and timelines. Data from the Demographic and Health Survey and the Health Management Information System (HMIS) were instrumental in estimating adherence rates and service utilization, yet their quality and accuracy remain variable. This underscores the need for strengthened data systems in Nepal to better support research, quantification exercises, and supply chain management for scaling up MMS.

Some potential challenges and limitations for the NAMASTE-MMS study include difficulties with recruitment of pregnant women, challenges with measurement of outcomes, and generalizability of findings. The trial relies on successfully recruiting a sufficient number of pregnant women in the sampled clusters; it is possible, however, that there may be fewer women at 12 to 13.9 weeks gestation or fewer reporting to the health facilities for ANC during this window than estimated from existing data. Although adherence is being measured via pill count, it also relies on an accurate recording of pills received and accurate reporting of whether any pills were lost, given away, or otherwise missing for reasons other than individual consumption; recall and other types of reporting bias thus may generate errors across the three arms. Finally, the findings are specific to women in the 12 to 13.9 weeks gestational window who sought ANC at a government health facility in one of Nepal’s seven provinces and surveyed throughout their pregnancy about supplementation adherence and acceptability; thus, the findings may not be fully generalizable beyond this context,

To generate comprehensive evidence to inform the feasibility of transitioning from IFA to MMS, complementary studies—including qualitative assessments with healthcare providers, pregnant women, and their families—will explore factors influencing adherence and acceptability, as well as the operational feasibility of MMS scale-up in Nepal. These findings will provide policymakers and program planners with actionable insights for implementing MMS at scale.

Globally, the NAMASTE-MMS study will contribute to the growing body of literature on MMS adherence and acceptability. The findings could support Nepal in an evidence-based adoption of MMS for antenatal and postnatal care and inform similar transitions in other low- and middle-income countries.

## Data Availability

Helen Keller will have access to the final trial dataset. Data sharing agreements can be made with other parties who request data for further analyses.

## Abbreviations

ANC: Antenatal care
c-RCT: Cluster-randomized controlled trial
ECF: Eleanor Crook Foundation
FCHV: Female Community Health Volunteer
FWD: Family Welfare Division
GEE: Generalized estimating equations
IFA: Iron and folic acid
MoHP: Ministry of Health and Population
MMS: Multiple micronutrient supplementation
NAMASTE-MMS: Non-inferiority Assessment of Maternal Adherence to Supplements, a Trial on the Effects of Multiple Micronutrient Supplementation Multiple Micronutrient Supplementation
NHRC: Nepal Health Research Council
ODK: Open Data Kit
PNC: Postnatal care
TCC: Trial Coordination Committee
TSC: Trial Steering Committee
